# Can Robotic Arm‐assisted Total Knee Arthroplasty Remain Cost‐effective in Volume‐based Procurement System in China? A Markov Model‐based Study

**DOI:** 10.1111/os.14078

**Published:** 2024-05-01

**Authors:** Zhuo Zhang, Yang Luo, Jing Zhang, Chong Zhang, Xin Wang, Jiying Chen, Wei Chai

**Affiliations:** ^1^ Department of Adult Reconstruction and Joint Replacement, Senior Orthopedic Department, Fourth Medical Center Chinese PLA General Hospital Beijing China; ^2^ Orthopedic Department, First Medical Center Chinese PLA General Hospital Beijing China; ^3^ Yunnan Baiyao Group Medicine Electronic Commerce Co., Ltd. Kunming China

**Keywords:** Cost‐effectiveness analysis, Markov decision model, Robotic arm‐assisted, Total knee arthroplasty

## Abstract

**Objective:**

The volume based procurement (VBP) program in China was initiated in 2022. The cost‐effectiveness of robotic arm assisted total knee arthroplasty is yet uncertain after the initiation of the program. The objective of the study was to investigate the cost‐effectiveness of robotic arm‐assisted total knee arthroplasty and the influence of the VBP program to its cost‐effectiveness in China.

**Methods:**

The study was a Markov model‐based cost‐effectiveness study. Cases of primary total knee arthroplasty from January 2019 to December 2021 were included retrospectively. A Markov model was developed to simulate patients with advanced knee osteoarthritis. Manual and robotic arm‐assisted total knee arthroplasties were compared for cost‐effectiveness before and after the engagement of the VBP program in China. Probability and sensitivity analysis were conducted.

**Results:**

Robotic arm‐assisted total knee arthroplasty showed better recovery and lower revision rates before and after initiation of the VBP program. Robotic arm‐based TKA was superior to manual total knee arthroplasty, with an increased effectiveness of 0.26 (16.87 *vs* 16.61) before and 0.52 (16.96 *vs* 16.43) after the application of Volume‐based procurement, respectively. The procedure is more cost‐effective in the new procurement system (17.13 *vs* 16.89). Costs of manual or robotic arm‐assisted TKA were the most sensitive parameters in our model.

**Conclusion:**

Based on previous and current medical charging systems in China, robotic arm‐assisted total knee arthroplasty is a more cost‐effective procedure compared to traditional manual total knee arthroplasty. As the volume‐based procurement VBP program shows, the procedure can be more cost‐effective.

## Graphical abstract

As the volume based procurement (VBP) program initiated, cost‐effectiveness of Robotic arm‐assisted total knee arthroplasty (RA‐TKA) remains unclear in China. The study compared the cost‐effectiveness of RA‐TKA with conventional manual TKA in a Markov decision model. RA‐TKA remains cost‐effective before and after the initiation of VBP program.

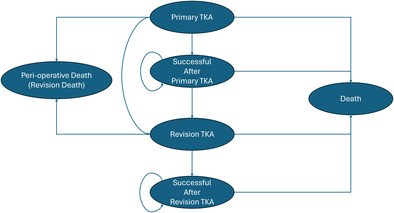

## Introduction

Total knee arthroplasty (TKA) has been proven to be an excellent option for advanced arthropathies, with considerable functional outcome and long‐term survivorship. However, some patients still complain of pain, instability, and discomfort after TKA for unknown reasons, which required evaluations for revision procedures. To address this, fine‐tune TKA surgical techniques have been developed to optimize the position and alignment of TKA components. Robotic arm‐assisted devices have been introduced recently, which may provide more accurate bone cut and more precisely measured alignments.

Previous studies have demonstrated that, better patient‐reported outcomes can be achieved by using robotic arm‐assisted devices during TKA procedure, as well as better surrounding soft tissue protection and decreased length of stay.^1^, ^2^, ^3^, ^4^, ^5^ On the other hand, robotic arm‐assisted surgeries may increase the total cost for each individual, with additional preoperative CT scans and special device‐related charges required by the robotic system, as well as prolonged surgical time, more complicated room settings and complications related to pin tracks, including intra‐and postoperative fractures.^6^, ^7^, ^8^ Despite these additional charges, the cost‐effectiveness of robotic arm‐assisted TKA (RA‐TKA) has been approved by several studies in different analysis models.^9^, ^10^, ^11^, ^12^ As robotic assisted TKA become more popular in China,^13^, ^14^ and the proportions of total cost are quite different in China and other countries, few studies have focused on the cost‐effectiveness of RA‐TKA in China.^15^ Furthermore, a volume‐based procurement (VBP) program was started in the end of April 2022 in China. The proportions of total cost of TKA have changed significantly, which may bring uncertainty to cost‐effectiveness of RA‐TKA. In the program, the cost of prosthesis decreased by up to 90%, while additional costs would be charged for robotic surgeries, including robotic‐related disposables and services.

The purpose of our study was to investigate the cost‐effectiveness of robotic arm‐assisted TKA *versus* conventional TKA in patients with knee osteoarthritis, before and after the implementation of the VPB program in China.

## Methods

### Decision Model

**FIGURE 1:**
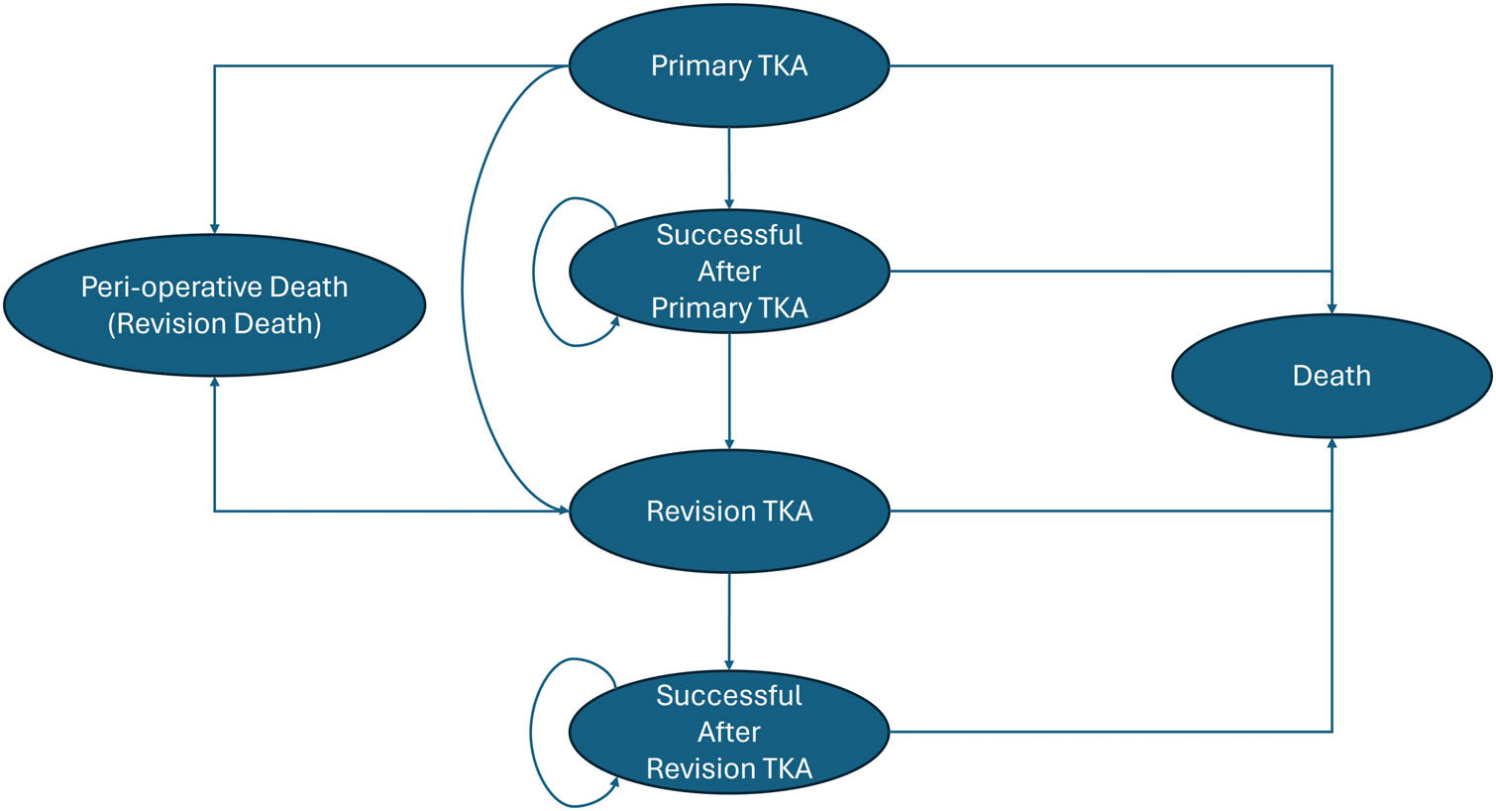
Visualization of the Markov model. A transition between phases in every 1‐year interval.

A Markov model was developed to simulate patients with advanced knee osteoarthritis who would be treated with traditional manual TKA (m‐TKA) or RA‐TKA. We constructed a time‐dependent transition between specific postoperative health states (Figure 1). The transition between health states were determined by transition probabilities. Each health state was defined by quality of life (QOL) values and costs.

There were several general assumptions made before the engagement of the model. First, yearly mortality rates are procedure‐specific, which will not differ between traditional TKA and RA‐TKA. Second, patients in this model underwent one revision surgery at most, of which the reason for revision excluded infections in any form. Afterward, the patients were assumed to be in “successful after revision TKA” state until death. Last but not least, costs except for implants in primary TKA procedures before and after VBP remained unchanged. As the model proceeded, patients experienced QOLs in each state would summate into quality‐adjusted life‐years (QALYs).

### Quality‐adjusted Life Years

Quality‐adjusted life years (QALYs) were used to represent the quality of life of patients in each group. These were determined based on previously published literatures. A well‐functioning TKA would receive a health utility of 0.840 and 0.857 for traditional and robotic arm‐assisted TKA, respectively.^10^, ^11^, ^16^, ^17^ We assumed that the health state for revision would be the same for traditional and RA‐TKA, which would receive a utility of 0.775.^10^, ^17^ Per standard Markov methodology, all QALYs were discounted at an annual rate of 3%. QALYs were calculated over a 20‐year period, based on the long‐term evidence of implant survivorship after TKA.

### Costs

Costs of each procedure were based on the previous inpatient data of our institute from 2019 to 2021. In order to assure consistency of costs, cases with prosthesis provided by the same manufacturer in the same surgical team were selected, as perioperative protocols and implant selection might differ among different surgical groups. Thirty‐seven primary TKAs were included in the analysis, of which 20 were performed before VBP and the other 17 were performed afterward. Fourteen revision cases were included as well.

As the difference of proportions in the total cost focused on the price of implants and robotic procedure related charges, other costs would remain unchanged before and after the initiation of the VBP Program, which would be demonstrated as medical service charges in our study. Costs of revision surgeries were calculated separately, based on previous data. As mentioned before, one revision surgery was assumed for each case which required revision, and debridement and implant retention (DAIR) or staged revision for prosthetic joint infections were not considered. Before the VBP initiation, cost of implant was 48,000 CNY per set, while surgery related charge was 3756 CNY for each patient. Price of implant decreased to 3439 CNY per set after the initiation of VBP. Meanwhile, charge of robotic surgery increased by 8000 CNY. Charges of preoperative CT scans for robotic‐surgical planning kept unchanged before and after VBP initiation, which was 600 CNY for each patient who would undergo an RA‐TKA procedure.

The total costs of m‐TKA and RA‐TKA before VBP were 71,607 and 72,207 CNY, respectively. and those after VBP were 27,046 and 35,646 CNY, respectively. Total cost for a revision procedure was 122,516 CNY, which did not change despite the VBP engagement. Parameters including costs used in the model are shown in Table 1. As the revision rate due to mechanical failure of manual TKA in China was similar to those reported in previous studies,^18^ and no confidential reports of revision rate of robotic TKA was available, the annual revision rates of m‐TKA and RA‐TKA were set to be 1.50% and 0.60%, respectively.

**TABLE 1:** Parameters used in Markov decision model

Variables in Markov Model	m‐TKA	RA‐TKA	Revision TKA
Transition probabilities			
Mortality	0.10%	0.10%	0.27%
Annual revision rates	1.50%	0.60%	‐
QALYs	0.840	0.857	0.775
Disutility	−0.1	−0.1	−0.2
Costs (CNY)			
Surgical procedure			
Before VBP	3756	3756	6612
After VBP	3756	11,756	6612
Implant			
Before VBP	48,000	48,000	51,834
After VBP	3439	3439	51,834
Robotic surgery‐related costs (including engineering services & consumptions)			
Before VBP	0	600 (CT scans)	‐
After VBP	0	8600	‐
Medical services			
Before VBP	19,851	19,851	32,677
After VBP	19,851	19,851	32,677
Total costs			
Before VBP	71,607	72,207	122,516
After VBP	27,046	35,646	122,516
Willingness‐to‐pay (WTP) Threshold (CNY): 242,928 CNY			

Abbreviations: CNY, Chinese Yuan; mTKA, manual total knee arthroplasty; QALY, quality adjusted life year; RA‐TKA, robotic arm‐assisted total knee arthroplasty; VBP, volume‐based procurement.

### Analysis

TreeAge Pro 2022 ver.1.1 (TreeAge Software, Williamstown, MA, USA) was used to simulate the Markov decision procedure. QALYs and costs were organized into an incremental cost‐effectiveness ratio (ICER), which represents the difference in costs divided by the difference in QALYs among different procedures. ICERs were evaluated with willingness‐to‐pay thresholds (WTPs). As the gross domestic product (GDP) *per capita* of 2022 was 85,698 CNY in 2022, the WTP in our model was defined as three times of GDP *per capita*, which was calculated as 257,094 CNY.^19^

One‐ and two‐way deterministic sensitivity analyses were performed on all transition probabilities and QALYs in our model, varying them from 0 to 1 to determine sensitive parameters that would shift the overall results of our model. Sensitivity analysis of the annualized per‐case robotic costs in this model were performed to determine the number of cases needed to treat for a robotic‐assisted TKA to be cost‐effective to an institution before and after VBP.

To ensure the interpretability and transparency of the reported content, and make the research evidence better served the medical decision‐making, an updating reporting guidance for health economic evaluations was followed.^20^

## Results

### Probability Analysis

**FIGURE 2:**
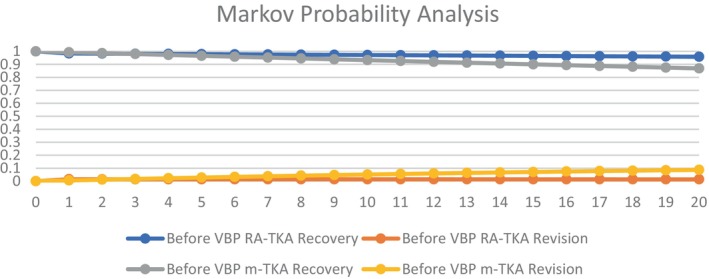
Probability analysis for primary total knee arthroplasty (TKA) before volume‐based procurement (VBP).

**FIGURE 3:**
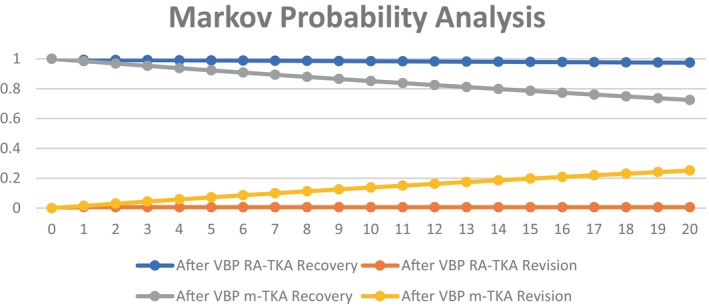
Probability analysis for primary total knee arthroplasty (TKA) after volume‐based procurement (VBP).

Probability analysis in our model demonstrated differences of recovery and revision rates for m‐TKA and RA‐TKA. RA‐TKA showed better recovery and lower revision rates before and after initiation of VBP (Figures 2 and 3).

### Cost‐effectiveness of m‐TKA and RA‐TKA

**FIGURE 4:**
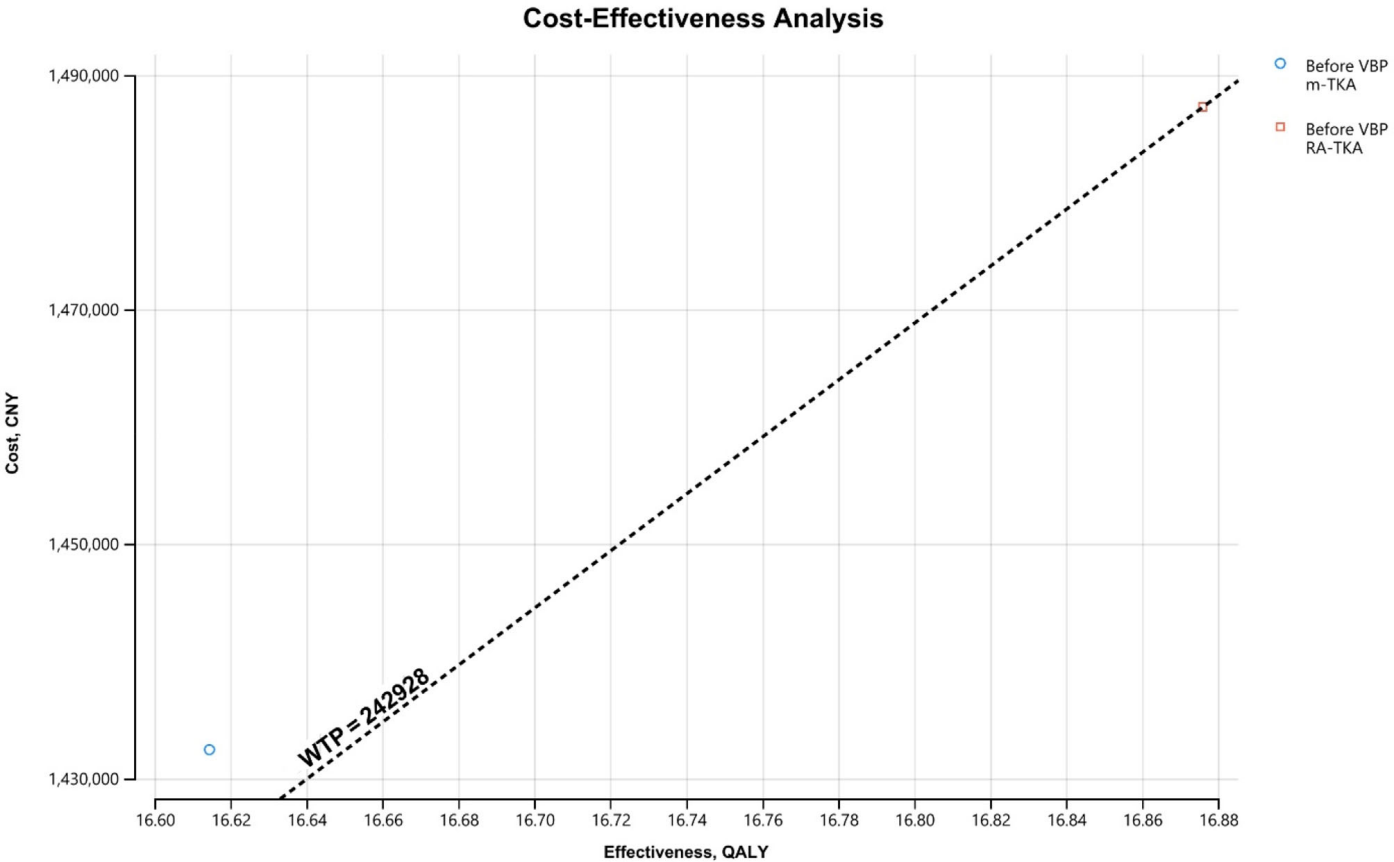
Cost‐effectiveness analysis showed better cost‐effectiveness for RA‐TKA before volume‐based procurement (VBP).

In our time‐dependent model, we calculated the total costs, QALYs and cost‐effectiveness of m‐TKA and RA‐TKA, respectively. Total costs of m‐TKA and RA‐TKA were 1,432,129 CNY and 1,485,794 CNY, respectively. Manual TKA gained 16.61 QALYs, while RA‐TKA gained 16.87 QALYs before the initiation of VBP. RA‐TKA showed better cost‐effectiveness (Table 2, Figure 4).

**TABLE 2:** Cost‐effective analysis for m‐TKA and RA‐TKA before VBP

Surgical procedure	Total cost (CNY)	QALY	Increased cost‐effectiveness	Increased cost	Increased effectiveness
m‐TKA	1,432,129	16.61	‐	‐	‐
RA‐TKA	1,485,794	16.87	209,665.4	53,665.15	0.26

Abbreviations: CNY, Chinese Yuan; m‐TKA, manual total knee arthroplasty; QALY, quality adjusted life years; RA‐TKA, robotic‐arm assisted total knee arthroplasty; VBP, volume‐based procurement.

**FIGURE 5:**
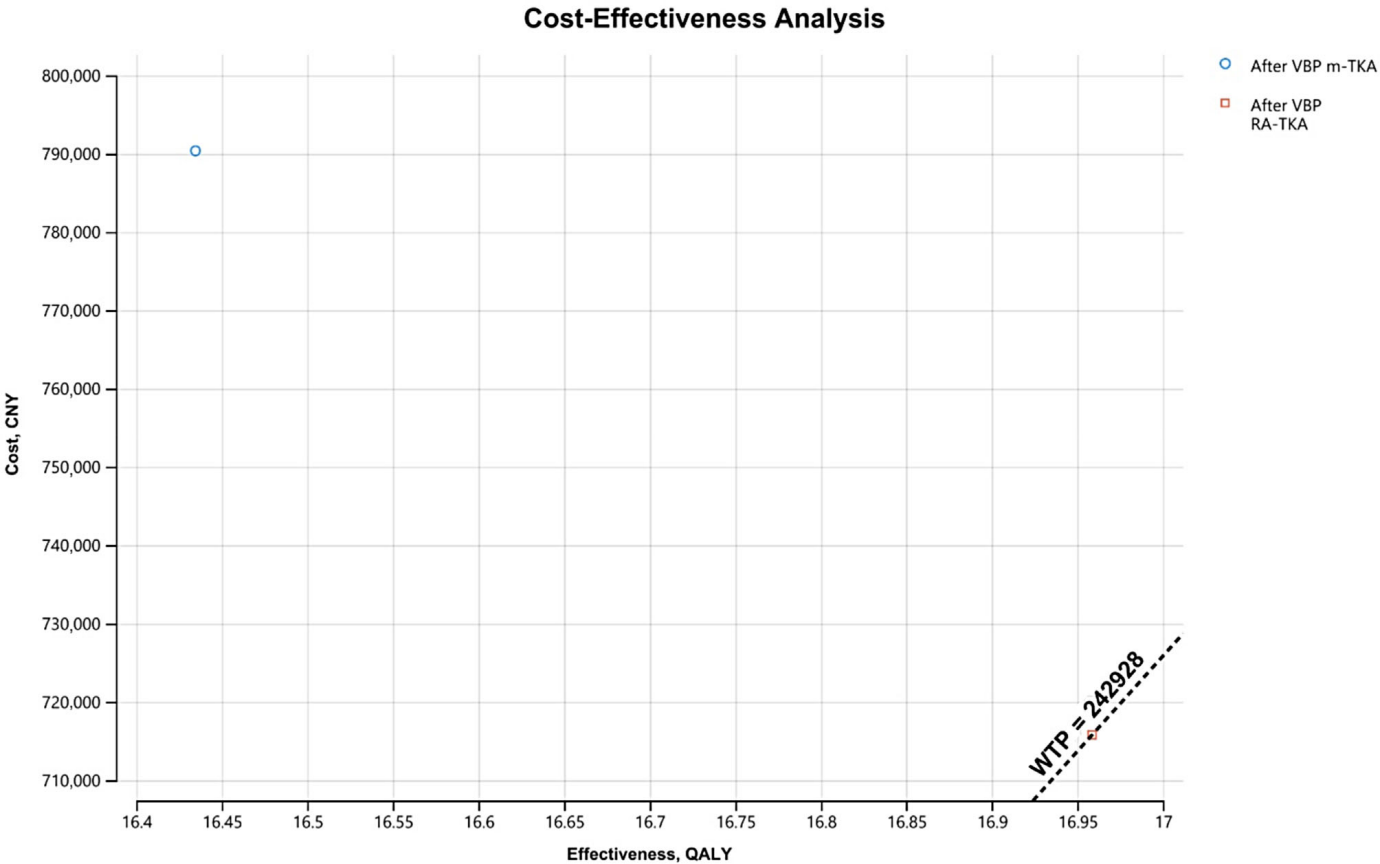
Cost‐effectiveness analysis showed better cost‐effectiveness for RA‐TKA after volume‐based procurement (VBP). The superiority of RA‐TKA decreased as the total costs decreased after VBP.

After VBP, total costs of m‐TKA and RA‐TKA were 790,354.9 CNY and 715,782.5 CNY, respectively. QALYs of the two procedures were 16.43 and 16.96, respectively. RA‐TKA remained more cost‐effective after the initiation of VBP (Table 3, Figure 5).

**Table 3:** Cost‐effective analysis for m‐TKA and RA‐TKA after VBP

Surgical procedure	Total cost (CNY)	QALY	Increased cost‐effectiveness	Increased cost	Increased effectiveness
m‐TKA	790,354.9	16.43	‐	‐	‐
RA‐TKA	715,782.5	16.96	142,334	74,572.41	0.52

Abbreviations: CNY, Chinese Yuan; m‐TKA, manual total knee arthroplasty; QALY, quality adjusted life years; RA‐TKA, robotic‐arm assisted total knee arthroplasty; VBP, volume‐based procurement.

**FIGURE 6:**
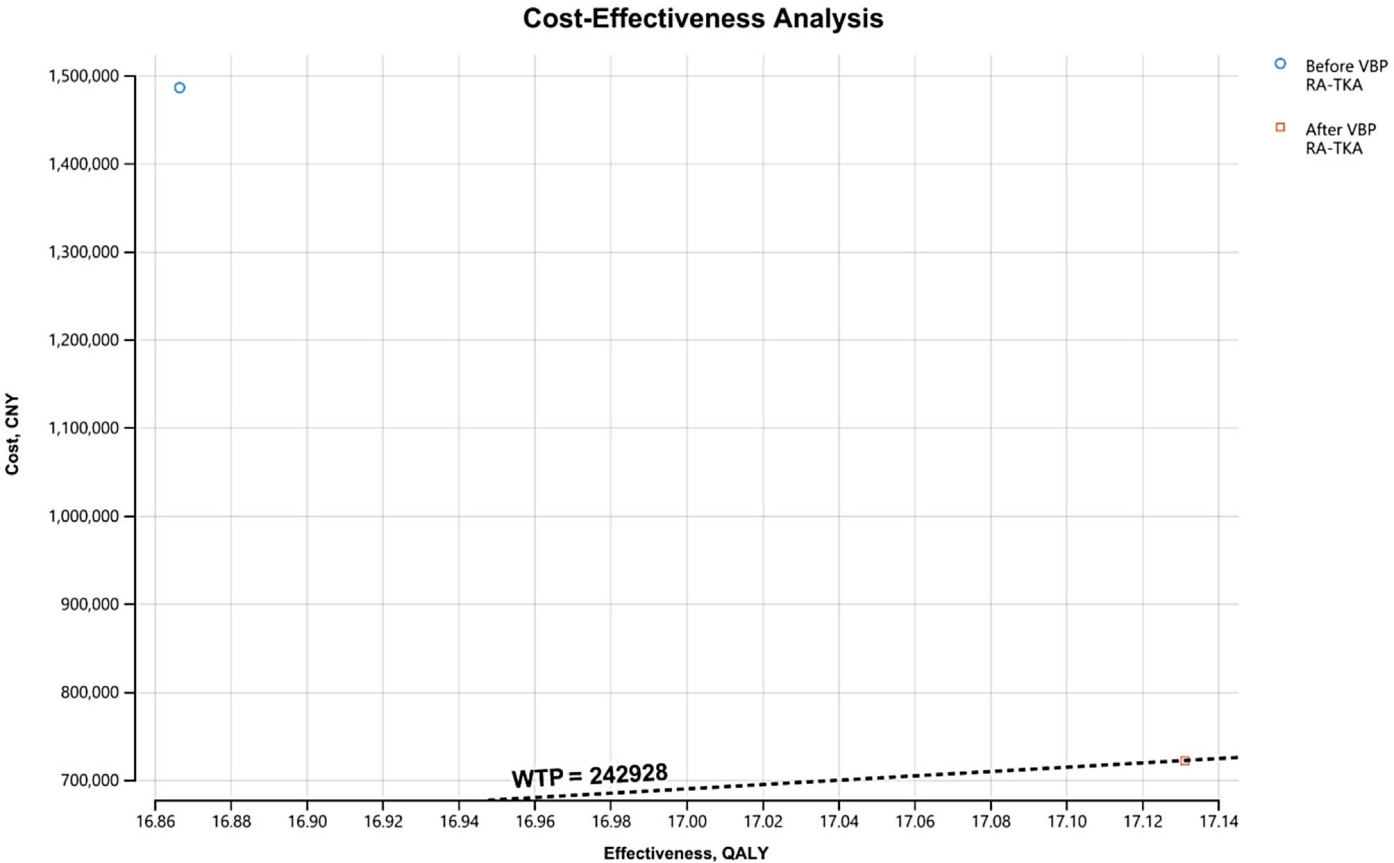
Cost‐effectiveness analysis comparing cost‐effectiveness of RA‐TKA before and after volume‐based procurement (VBP). The result showed better cost‐effectiveness for RA‐TKA after VBP.

For robotic‐arm assisted total knee arthroplasty, we performed another analysis to compare cost‐effectiveness of the procedure before and after the initiation of VBP. The total cost of RA‐TKA before VBP was 1,485,794 CNY, and the cost decreased to 721,588 CNY after VBP. QALY of the procedure increased from 16.89 to 17.13. Although the effectiveness increase was limited after VBP, the significantly decreased cost of the procedure made it more cost‐effective after the engagement of the national program (Table 4, Figure 6).

**TABLE 4:** Cost‐effective analysis for RA‐TKA before and after VBP

Surgical procedure	Total cost (CNY)	QALY	Increased cost‐effectiveness	Increased cost	Increased effectiveness
RA‐TKA before VBP	1,485,794	16.89	‐	‐	‐
RA‐TKA after VBP	721,588	17.13	2,887,006	−764,236	0.26

Abbreviations: CNY, Chinese Yuan; QALY, quality adjusted life years; RA‐TKA, robotic‐arm assisted total knee arthroplasty; VBP, volume‐based procurement.

### Sensitivity Analysis

**FIGURE 7:**
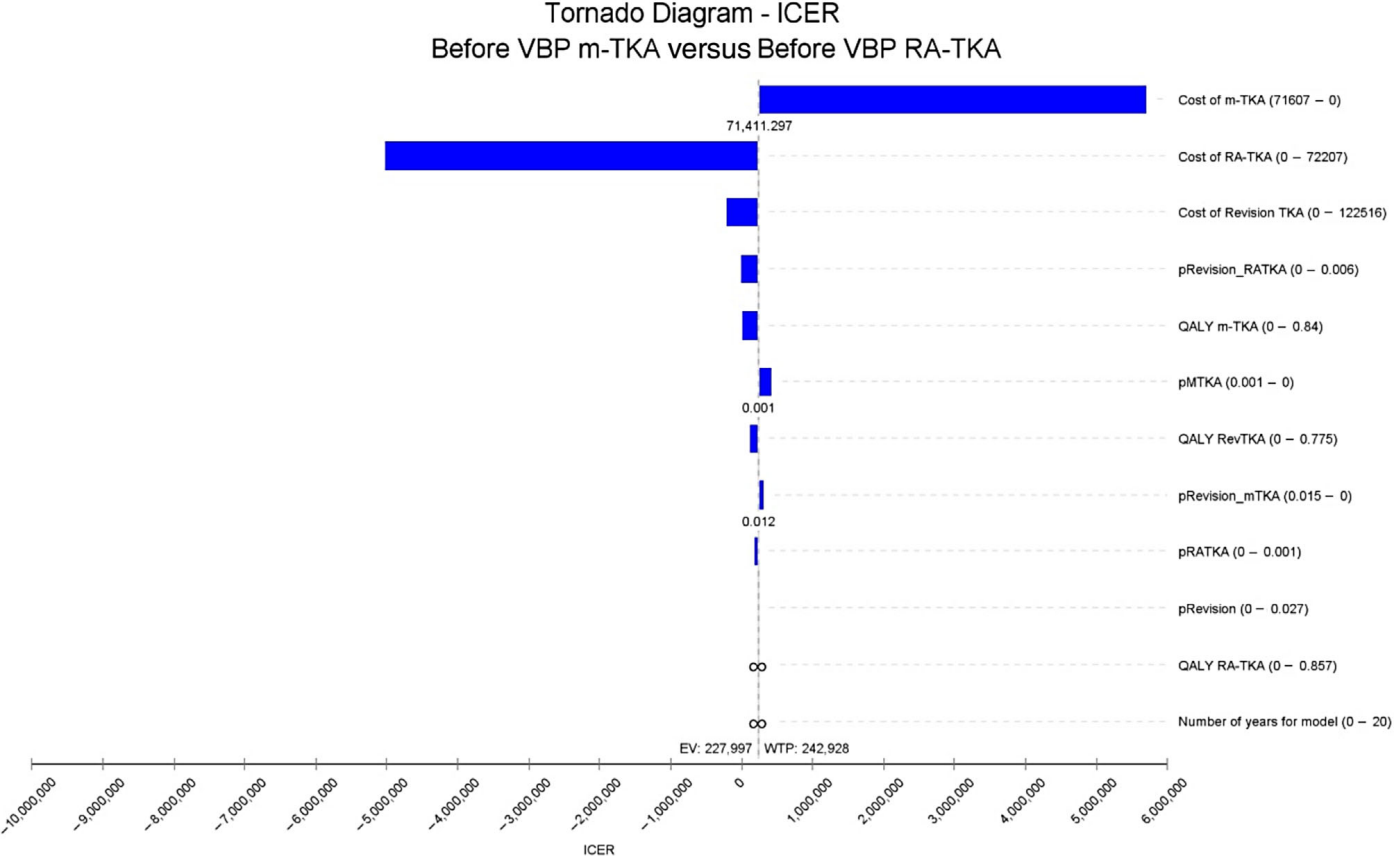
Tornado diagram demonstrating the most sensitive parameters which influence the percentage change in quality‐adjusted life‐years (QALYs) for manual total knee arthroplasty (m‐TKA) and RA‐TKA before VBP. The most sensitive parameters in this model were costs of m‐TKA and RA‐TKA.

**FIGURE 8:**
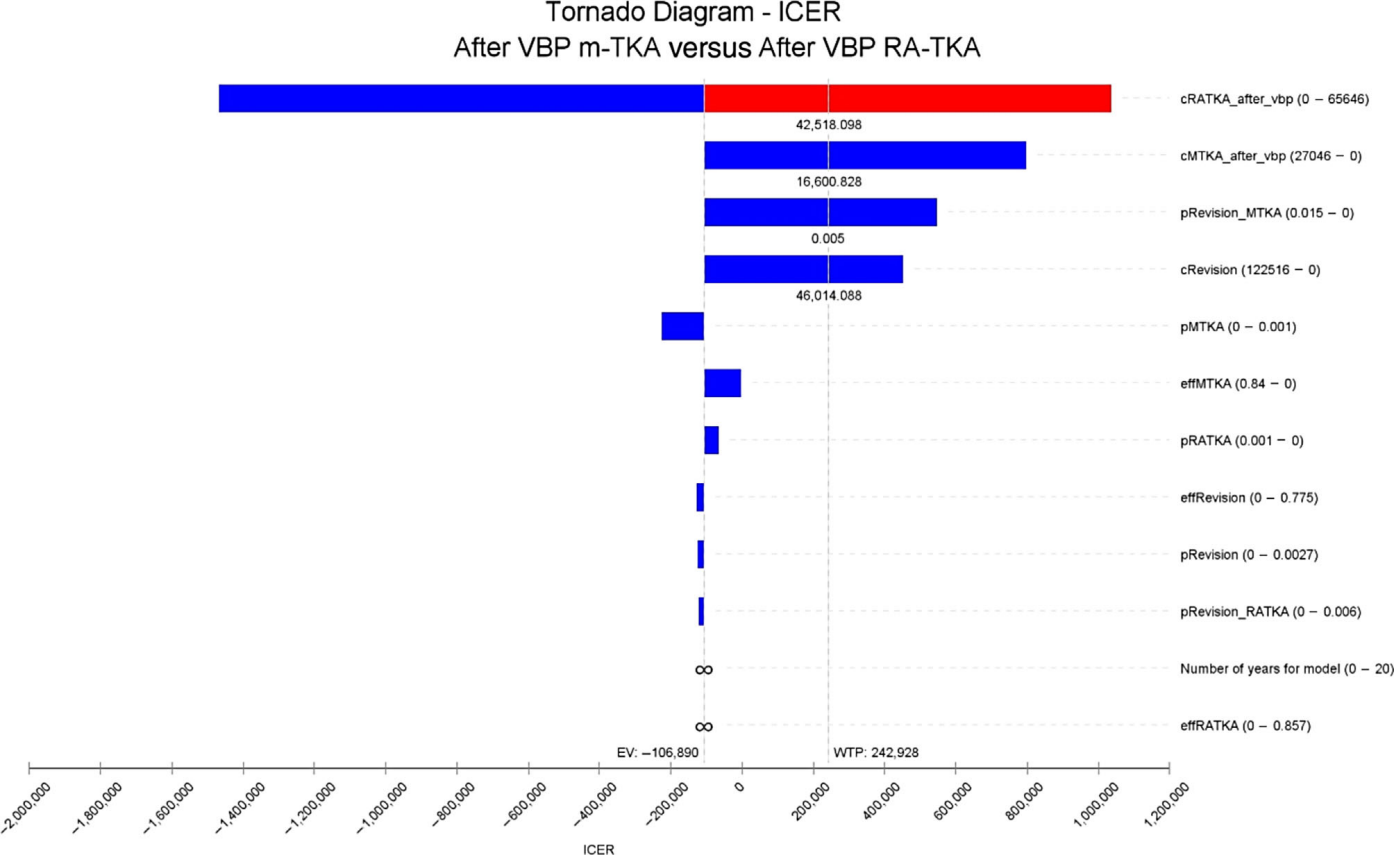
Tornado diagram demonstrating the most sensitive parameters which influence the percentage change in QALYs for manual total knee arthroplasty (m‐TKA) and RA‐TKA after volume‐based procurement (VBP). Although rankings and values of some parameters changed after VBP, the most sensitive parameters in this model were still costs of m‐TKA and RA‐TKA.

Results of the sensitivity analysis are demonstrated by the Tornado Charts in Figures 7 and 8. The most sensitive variables were the costs of m‐TKA and RA‐TKA. Although the total cost of each procedure decreased after initiation of VBP, costs remained the most sensitive parameters in our country.

**FIGURE 9:**
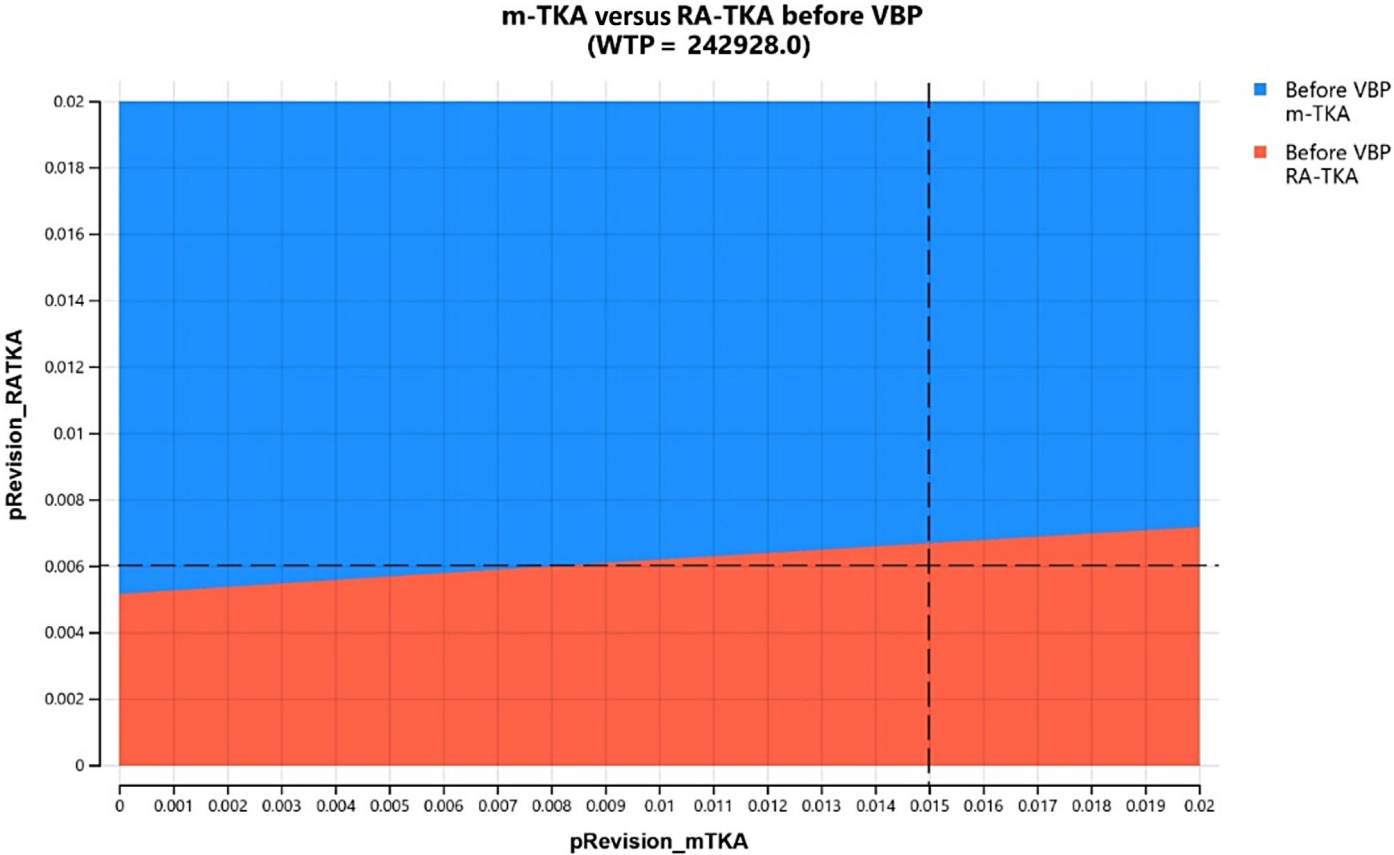
Two‐way sensitivity analysis for manual total knee arthroplasty (m‐TKA) and RA‐TKA after volume‐based procurement (VPB). At the base case of 1.5% for m‐TKA and 0.6% for RA‐TKA, RA‐TKA was the preferred procedure.

**FIGURE 10:**
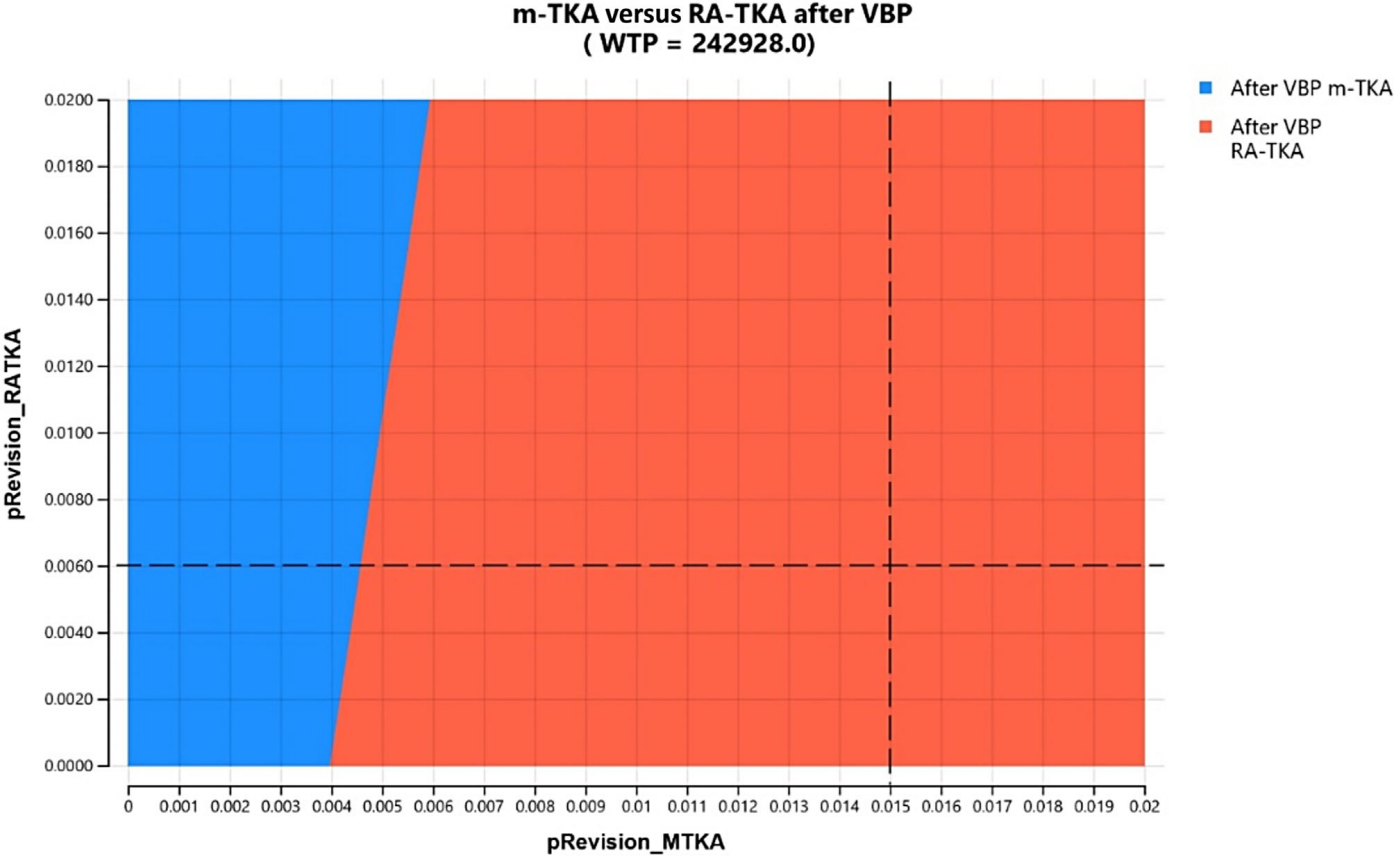
Two‐way sensitivity analysis for manual total knee arthroplasty (m‐TKA) and RA‐TKA after volume‐based procurement (VPB). At the base case of 1.5% for m‐TKA and 0.6% for RA‐TKA, RA‐TKA remained preferred.

Two‐way sensitivity analysis was performed as well. At the base case of 1.5% for m‐TKA and 0.6% for RA‐TKA, RA‐TKA was the preferred procedure before and after VBP (Figures 9 and 10).

## Discussion

### Cost‐effectiveness of RA‐TKA

The Markov model is widely used in medical research, including the evaluation of new technologies in surgical procedures, including innovations in joint replacements.^10^, ^11^, ^16^, ^21^, ^22^, ^23^ RA‐TKA may provide more accurate bone cut and implant position, as well as less invasive influence to surrounding soft tissue and better short‐term patient‐reported outcomes. Current literature has demonstrated that robotic arm‐assisted devices can improve cost‐effectiveness of total knee arthroplasties.^10^, ^11^ Potential decrease in rates of complications and revision surgeries applied may contribute to the superior cost‐effectiveness of RA‐TKA procedures. However, due to the different proportions of inpatient costs in China, of which costs of implants took a major part, cost‐effectiveness of such procedures may differ from that in Western countries.^15^ Thus, cost‐effectiveness analysis may have different results as well. Our study showed similar results, and no matter in previous or current Chinese procurement systems, RA‐TKA has been proven to be more cost‐effective comparing to traditional manual TKA.

The most sensitive parameters in our model were the costs of m‐TKA and RA‐TKA, while the utility of robotic‐assisted surgeries and the utility of revision procedure were reported to be most sensitive in the published literature in Europe and the US.^10^, ^11^ Again, the truth that costs of implants took major proportion in the total expense of each procedure altered the ranking of sensitive parameters.

### Influence of VBP to Cost‐Effectiveness of RA‐TKA

Another analysis comparing the cost‐effectiveness of RA‐TKA before and after VBP was applied. To our surprise, the results showed superior cost‐effectiveness in the current procurement system, even if the parameters other than implant cost and robotic surgery‐related charges were assumed to be unchanged. The significantly higher decrement of implant cost than the increment of robotic‐related charges may be responsible. This also indicated that, in previous and partial current medical charging systems in China, implant or medical consumptions still make a major contribution to the total cost.^15^

To our knowledge, this is the first study to investigate the cost‐effectiveness of RA‐TKA in the Chinese population and procurement system. With the initiation of the VBP program, costs of implants decreased by 90%, while other expense items remained unchanged. We assumed that the revision rates of m‐TKA and RA‐TKA remained unchanged during the engagement of VBP. However, the lower price of implants in the current procurement system may ease the decision of applying revision procedures to problematic TKA, which would increase the revision rates, and change the results. Moreover, as the VBP program proceeds, there would be uncertainties. Policy adjustment in the medical paying system, price changes of other charging items such as medical service or medication, additional charges for robotic‐related consumptions such as trackers and fixation pins, as well as services and governmental administration of innovative surgical techniques can all be the uncertainties aforementioned.

### Strengths and Limitations

Our study is a cost‐effective analysis based on Markov decision model. Retrospective data were used in the model, and results of the analysis were based on assumptions. As all assumptions were based on previous data and published literature, it may present bias to real‐world situations. Despite this, the study still represents the trend of cost‐effectiveness of the procedure.

Our study had several limitations. First, the model we used in the study was based on several assumptions. As the nature of the model itself, results of the model were too sensitive to any alternations of variations included in the model. The data used in the decision model was collected at a single institute during limited time duration. Variation does exist among hospitals in different regions. Results from real‐world studies would be far more convincing than those from such an analysis model. Second, a single brand implant was taken in the model with fixed prices before and after the initiation of VBP, while the results may only represent the cost‐effectiveness of such implant using manual or robotic arm‐assisted techniques. Costs other than implants and robotic‐related charges were assumed unchanged before and after the application of the national policy. When different variations were taken into the model, results may reverse with changes of implant prices, charges on medical services and robotic‐related issues. Influence of regional medical insurance policy was not considered as well. For a more comprehensive cost‐effectiveness assessment of a novel technique, more detailed data from different institutes may be necessary. Last but not the least, the model was set up based on current data, including revision rates, charging items, and charging values. Uncertainty exists under the background of the VBP application, in which costs of implants and charges of medical services may change in the future. Long‐term analysis for cost‐effectiveness should include dynamic data collection for a more accurate evaluation.

## Conclusion

In conclusion, according to current data we collected, RA‐TKA is a more cost‐effective procedure comparing to traditional manual TKA in China. As the volume‐based procurement program is applied, the procedure can be more cost‐effective. However, the conclusions were based on some assumptions and were limited by parameters in the model. Caution is advised when applying the cost‐effectiveness results in different regions in China. For a more comprehensive evaluation, more detailed and dynamic data should be collected, which may require the establishment of a high‐quality joint registry system in China and initiation of a real‐world study.

## Conflict of Interest Statement

The authors declared no conflicts of interests.

## Author Contributions

Z.Z. and Y.L. contributed equally to the study, and they should be considered co‐first authors of the manuscript. Z.Z.: conceptualization, methodology, writing of the original draft, and project administration and supervising. Y.L.: shared works in writing and data analysis in the manuscript and led in reviewing and editing of the manuscript. J.Z. and X.W.: resource collection and data curation. C.Z.: software utilization and shared work in data analysis. J. C. and W.C.: leads i the conceptualization and monitoring of the project.
